# HIP^2^: An online database of human plasma proteins from healthy individuals

**DOI:** 10.1186/1755-8794-1-12

**Published:** 2008-04-25

**Authors:** Sudipto Saha, Scott H Harrison, Changyu Shen, Haixu Tang, Predrag Radivojac, Randy J Arnold, Xiang Zhang, Jake Yue Chen

**Affiliations:** 1 School of Informatics, Indiana University – Purdue University, Indianapolis, USA; 2 Indiana Center for Systems Biology and Personalized Medicine, Indiana University – Purdue University, Indianapolis, Indianapolis, USA; 3 Division of Biostatistics, Indiana University School of Medicine, Indianapolis, USA; 4 School of Informatics, Indiana University, Bloomington, USA; 5 Department of Chemistry, Indiana University, Bloomington, USA; 6 Department of Chemistry, University of Louisville, Louisville, USA; 7 Department of Computer & Information Science, Purdue University, Indianapolis, USA

## Abstract

**Background:**

With the introduction of increasingly powerful mass spectrometry (MS) techniques for clinical research, several recent large-scale MS proteomics studies have sought to characterize the entire human plasma proteome with a general objective for identifying thousands of proteins leaked from tissues in the circulating blood. Understanding the basic constituents, diversity, and variability of the human plasma proteome is essential to the development of sensitive molecular diagnosis and treatment monitoring solutions for future biomedical applications. Biomedical researchers today, however, do not have an integrated online resource in which they can search for plasma proteins collected from different mass spectrometry platforms, experimental protocols, and search software for healthy individuals. The lack of such a resource for comparisons has made it difficult to interpret proteomics profile changes in patients' plasma and to design protein biomarker discovery experiments.

**Description:**

To aid future protein biomarker studies of disease and health from human plasma, we developed an online database, HIP^2 ^(Healthy Human Individual's Integrated Plasma Proteome). The current version contains 12,787 protein entries linked to 86,831 peptide entries identified using different MS platforms.

**Conclusion:**

This web-based database will be useful to biomedical researchers involved in biomarker discovery research. This database has been developed to be the comprehensive collection of healthy human plasma proteins, and has protein data captured in a relational database schema built to contain mappings of supporting peptide evidence from several high-quality and high-throughput mass-spectrometry (MS) experimental data sets. Users can search for plasma protein/peptide annotations, peptide/protein alignments, and experimental/sample conditions with options for filter-based retrieval to achieve greater analytical power for discovery and validation.

## Background

A surge of interest in defining molecular biomarkers of health and disease from human plasma has recently emerged with the recent launch of the pilot Plasma Proteome Project (PPP) by the Human Proteome Organization (HUPO) [1]. The easy clinical access and processing of plasma samples, and the abundance of proteins as well as metabolites that may collectively define a person's health status, have made human plasma the top choice among bio-fluids for future clinical molecular diagnostic applications. The fluctuating nature of blood from different individuals, huge dynamic protein concentration ranges (up to 10^12^), and the protein detection limits of most MS platforms, have made the plasma proteome elusive to define. Many proteomics researchers even believe that the current "plasma proteome" observed by a single shotgun MS experiment is analogous to a stochastic sampling of the human proteome, with low run-to-run consistencies and inherent detection biases peculiar to each type of MS platform [2]. Even when used for healthy individual plasma, with multi-dimensional separations and advanced bioinformatics search software tools, proteins identified in different shotgun MS/MS plasma proteomics experiments are often inconsistent with each other except for the most abundant proteins. To overcome the poor coverage, potential bias, and complementary nature of each experimental measurement of the human plasma proteome, it is necessary for biomedical researchers to collect and assess all reliable publicly-available plasma protein data sets generated from different MS analytical and computational platforms for healthy individuals. A comprehensive integrated resource of the human plasma proteins for healthy individuals, currently missing in the field of clinical proteomics, would enable researchers to understand the basic constituents, diversity, and variability of the human plasma proteome. Such a resource would provide a high amount of comparative power for interpreting proteomics profile changes in patients' plasma, and may supplement or compensate for limitations and biases associated with the set of controls for a given study. It would also improve the ability for finding protein biomarkers that are known to occur in healthy human plasma for instances where a protein is differentially expressed in a patient sample related to the quantities observed in the study control.

Although multiple projects to profile the human plasma proteome have been attempted, including PeptideAtlas, GPMO, HUPO PPP, and several recent publications [3-6], an unaddressed need has been for a compiled, central repository structured to enable the stable retrieval, comparison and querying of results. The existing sources vary widely in terms of data set size, available user interface, experimental protocol or sample details, choices of protein identifiers, linking to peptide evidence from MS experiments, MS search software used, and extent of data annotation. This information needs to be compiled further and assembled for end users before they can consider incorporating human plasma proteome data into their studies. The largest single source of data is from an independently conducted experimental study that utilized a ion-mobility spectrometry (IMS) platform to chart the existence of 9,087 proteins based on 37,842 unique inferred peptide sequences, of which 2,928 proteins are high-confidence [6]. There is not however a web-based interface or other online resource for making this data widely available. The other sources of data we examined have some form of online presentation (aside from publication) and range in size from less than one thousand to over several thousand identified proteins. The Plasma Proteome Database (PPD) provides a web interface and is geared for providing detailed functional annotations of 3,778 distinct proteins based on data extracted from the literature, yet the PPD provides information on neither experimental protocol or associated MS-detected peptides used for protein identification [7]. HUPO PPP information consists of 3,020 proteins and 47,950 peptides along with experimental protocol information and is available to the public online, but the data is only accessible as flat files [8]. The Institute of Systems Biology (ISB) has surveyed and analyzed a comparably smaller set of data produced by 28 human plasma proteomics experiments, and has reported an approximate count of 960 proteins based on the 6,929 distinct observed peptides in their web-interfaced PeptideAtlas database [3]. Another resource, providing evidence for human proteomics based mainly on data from HUPO PPP, is hosted by the Global Proteome Machine Organization (GPMO). An important feature of the GPMO database is that it provides annotated information to assist with the difficult process of validating peptide MS/MS spectra and patterns of protein coverage [4]. GPMO also includes data from non-human organisms such as cats, guinea pigs, rabbits, unicellular eukaryotes like yeasts, as well as a number of prokaryotic organisms.

By gathering the protein and peptide data used to characterize the proteome of a healthy individual, we made an attempt to develop a resource that presents a comparative baseline of plasma proteomics results against which proteomic data from patients with diseases such as cancer, neurodegenerative diseases, metabolic diseases, and other genetic disorders may be studied. In this effort, we define "healthy" or "normal" as human adults without major known life-threatening diseases, genetic diseases, HIV, or inflammation at the time of blood drawing (a slightly more stringent variation than the HUPO definition in Omenn *et al*. [5]). On these premises, we developed an integrated database HIP^2 ^(Healthy Human Individual's Integrated Plasma Proteome) by compiling all of the existing experimental data performed on healthy individual samples, and creating a web-based interface to aid the many upcoming projects of protein biomarker studies of health and disease [9]. With HIP^2^, clinical samplings of patient plasma may be better compared to random or non-random aspects of overlap with the reported set of healthy human plasma proteins.

## Construction and content

**Figure 1:**
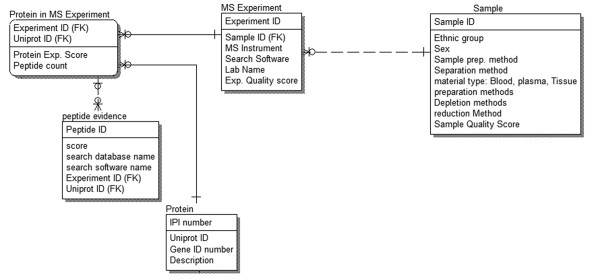
**Relational data model schema for the table**. Protein, peptide evidence, MS Experiment and sample.

We collected 3,020 proteins and 47,950 peptides from HUPO PPP in text format [5]; 9,087 proteins and 37,842 peptides from David Clemmer's group (DCG) recent publication supplemental material (in PDF format) [6]; 788 proteins and 6,039 peptides from PeptideAtlas (through successive querying of the web interface) [3] and 1,175 proteins from Leigh Anderson's group (LAG) (in PDF format) [10]. HUPO contain two datasets: i) a dataset consisting of 3,020 proteins, and ii) a dataset consisting of 9,504 proteins. In our case, we included the high confidence core dataset of 3,020 proteins, where there are two or more peptide hits. HUPO datasets come from 35 collaborating laboratories, and for the single-peptide hits in the second data set, the number of laboratories can itself introduce high amounts of noise from diverse laboratory procedures and MS platforms, potentially acting to reduce value of the data resource for large-scale interpretation. In contrast, the single-peptide hits used in HIP^2 ^come from a single laboratory source, DCG. The uniformity attributable to this single laboratory source (and single MS platform) merited inclusion of all 9,087 proteins coming from this group. Data from the PeptideAtlas database were also included, but was filtered to only include those with publicly available experimental details. The integration of data from different resources is a non-trivial bioinformatics task, because the data sets are not standardized syntactically or semantically. To resolve syntactic incompatibility, we wrote perl programs and converted all the original data sets into comma-delimited files, and loaded them into an Oracle 10G database server as staging tables. To resolve semantic incompatibility, we developed a data model first as shown in Fig. 1, and then converted all the incompatible identifiers to standard IPI accession numbers [11], using a mapping table downloaded from BioMart [12]. HUPO PPP and PeptideAtlas proteins are indexed with IPI accession numbers, whereas the other sources of data are indexed with other types of identifiers such as Swiss-Prot accession numbers and RefSeq IDs. Correspondence with other identifiers was achieved for some of the text-based sources by parsing the sequence header data inside FASTA files, and the integrity of the mapping has been validated through comparisons of referenced sequences. A subset of 247 proteins from the LAG data set did not have correspondence with IPI accession numbers, and this subset was excluded from comparisons between the four sources of data. The set of 788 proteins from PeptideAtlas represents a subset of unique proteins from a larger set of 960 proteins. The data from DCG contains multiple splice variants labeled by numbers appended to Swiss-Prot identifier strings, and the first enumerated variant was found to map to the IPI accession number in all cases. Table 1 presents a union built from the four sources of data used in HIP^2^, and also categorizes proteins in terms of MS-based detection platform and search software.

**Table 1:** Summary of HIP^2 ^database. The numbers of peptides and proteins represent unique entries that are the union of multiple subjects, possibly from different ethnic groups.

Source	Platform	Peptides	Proteins	Search Software
**HUPO PPP **(3,020 proteins and 47,950 peptides)	ESI-MS/MS_DECA	712	348	SEQUEST
	ESI-MS/MS_DECAXP	5796	2149	SEQUEST
	ESI-MS/MS_LCQ	1818	427	SEQUEST/SONAR
	ESI-MS/MS_QSTAR	309	137	SEQUEST
	ESI-MS/MS_QTOF	5078	573	MASCOT
	ESI-MS/MS_QTRAP	195	51	MASCOT
	MALDI_MS/MS_QSTAR	384	60	SEQUEST/MASCOT
**David Clemmer's group **(9,087 proteins and 37,842 peptides)	IMS_MS/MS_TOF	35781	9,087	MASCOT
**PeptideAtlas **(788 proteins and 6,039 peptides)	ESI-MS/MS_DECA	260	110	SEQUEST
	ESI-MS/MS_DECAXP	317	159	SEQUEST
	ESI-MS/MS_LCQ	1101	263	SEQUEST
	ESI-MS/MS_QSTAR	14	14	SEQUEST
	ESI-MS/MS_QTOF	728	215	SEQUEST
	LC_MS/MS*	1153	250	SEQUEST
**Leigh Anderson's group **(1,175 proteins)	2DEMS & LC_MS/MS*	----	928	SEQUEST

*Not defined

**Figure 2:**
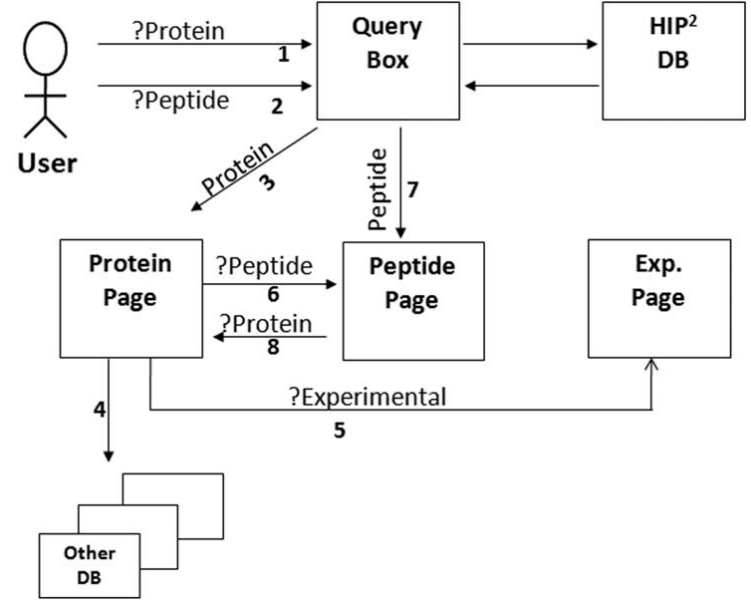
**The overall design of the HIP^2 ^database**. 1 = Query of protein "X" observed in plasma proteome; 2 = Query of peptide "Y" observed in plasma proteome; 3 = Output result of "X" protein page ; 4 = Link to external database; 5 = Link to experimental page of protein "X"; 6 = Link to the peptide page of the protein "X"; 7 = Output result of peptide "Y"; 8 = Iterative query from peptide page to search for other proteins associated with the peptide "Y".

We show the overall design of the database in Fig. 2. Queries from web site users are connected to a backend relational Oracle 10G database hosted at Indiana University's High-Performance Computing Facility. The web interface and database connectivity was implemented with PHP and perl. The HIP^2 ^web interface outputs results for user queries into three different types of web pages: protein pages, peptide pages, and experimental information pages. The database result pages are also linked to external web pages (see details on the "Help" section of the HIP^2 ^database home site). The protein information page provides information as to whether a protein is in the healthy human individual's plasma proteome, what function it has, what peptides can be mapped to the protein, and how the peptides are aligned according to trypsin digestion criteria [13]. The HIP^2 ^protein-peptide alignment map highlights potential trypsin cleavage sites in red. Identified peptides are displayed in green and are aligned underneath the corresponding sequence in the protein. The peptide information page provides peptide summaries, experimental evidence and the aforementioned peptide-protein alignment maps. Peptide summaries provide information about the amino acid sequence length, a link to the PeptideAtlas database for each peptide, and a link to the Statistical Analysis of Protein Sequences server [14] for researchers interested in peptide compositional patterns. The experimental information page contains additional details about the experiment whenever disclosed including the human subject's ethnic group, gender, sample preparation method, protein separation, material type, peptide separation, depletion method, and reduction method (treatment of indoacetamide). HIP^2 ^also includes information on material type to distinguish plasma and serum. We plan to develop an automated web interface for future data contributions, using a standard data submission format that is compliant with the upcoming MIAPE proteomics standards [15].

## Utility and discussion

The HIP^2 ^database provides protein biologists and clinical biomedical researchers with a new gateway for exploring proteins from the human proteome with peptide-level evidence found in plasma. The basic questions that the HIP^2 ^database helps biological researchers answer includes "whether a protein may be found in human plasma" and "how likely or easily it is for a protein to be observed in healthy human plasma with mass spectrometry." The HIP^2 ^database allows its users to assess the confidence of identifying plasma proteins in "normal" plasma MS proteomics experiments by examining such evidence as the number of matched peptide hits, data sources covered, MS experiments observed, types of MS platforms, and search software used. The protein and peptide sequence information also enables the user to examine peptide evidence that may be mapped to different gene splice variants and protein isoforms. Partial protein trypsin digestions can be evaluated based on multiple peptide to protein alignment information presented in the database. The overall quality of digested peptide mapped to proteins can be further used by mass spectrometry data analysts to assess different performance of MS proteomics platforms or samples. A typical example in the HIP^2 ^database of a protein-peptide sequence comparison is how the peptide sequence 'KQSAGLVLWGAILFVAWNALLLLFFWTRPAPGRPPSVSALDGDPASLTR' is present in two proteins, IPI00000138 and IPI00179044, and aligns with the same sites of trypsin cleavage (as shown in Fig. 6). In the case of protein IPI00000138, evidence for the protein was found in three data sources, three MS experiments, three MS platforms, two MS search software and six mapped peptide sequences, whereas in the case of protein 'IPI00179044', there are not any experimentally proven peptides from MS results.

**Figure 3:**
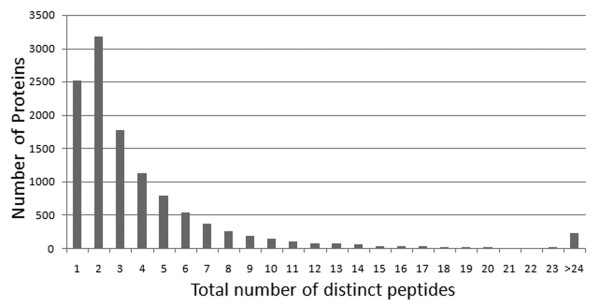
**Protein identification**. Number of proteins (Y axis) versus the number of distinct peptides used for the protein identification (X axis).

**Figure 4:**
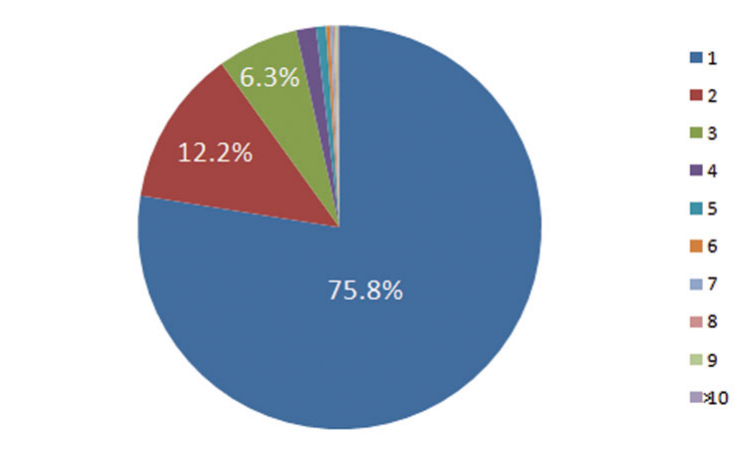
**Total proteins categorized by the number of platforms that identify them**. Numbers in the legend refer to the number of platforms.

**Figure 5:**
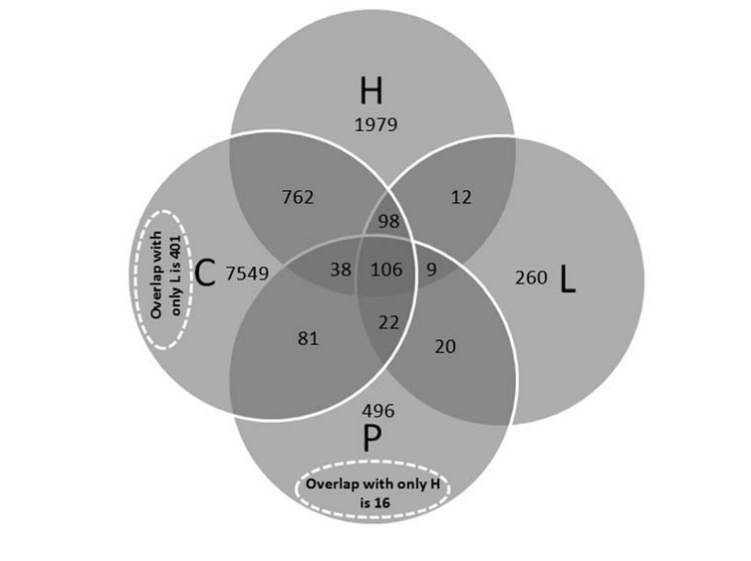
**Overlapping of plasma proteins identified from different sources**. C = Clemmer's group; H = HUPO PPP ; L = Leigh Anderson's group; P = PepAtlas data source.

The HIP^2 ^database has been mapped to cover a significant portion of the human proteome, which can be retrieved by user queries of plasma proteins or peptides, with full MS experimental context and detailed peptide-to-protein mapping relationships. As of the latest version (Feb 2008), the HIP^2 ^database aggregates information from 14 different protein separation/MS analytical platforms, 63 different samples, and 6 different types of MS search software. The HIP^2 ^database provides associated identifications of 12,787 protein entries representing 11,588 unique proteins covering 17% of all 67,511 proteins in the human IPI database [11]. Over 77% of the proteins included are identified from two or more different peptide sequences as shown in Fig. 3, where identification is defined by both peptide detection and a protein sequence match. We observed that almost 24% of the proteins included have been identified from two or more experimental platforms as shown in Fig. 4, while only 106 proteins (<1%) are identified by all four sources, implicating substantial variation in protein detections caused by a number of factors including experimental parameters and subject-to-subject variation (Fig. 5). Finding detailed instances of experimental detection of proteins by experimentally proven peptides is necessary to evaluate future options for experimental detection. For biostatisticians, the HIP^2 ^database can be a supplemental resource for assessing the likelihood of observing different peptides of the same plasma proteins from different platforms. Our resource is also useful to computational proteomics researchers, since we enable navigation among various ranges of association between a peptide sequence and a candidate protein sequence; this information may be valuable for assigning probabilistic confidence of all human proteins, whether or not they have been experimentally observed in human plasma [16]. The study of how search software is used in the experimental protocol, especially for how sequence alignment and locations of potential tryptic cleavage sites (highlighted by the HIP^2 ^interface) influence peptide detection, can now be directly addressed by the proteomics community. This online HIP^2 ^database can be a valuable source of information to biomedical researchers as they interpret proteomics profile changes in patients' plasma and pursue biomarker discovery.

**Figure 6:**
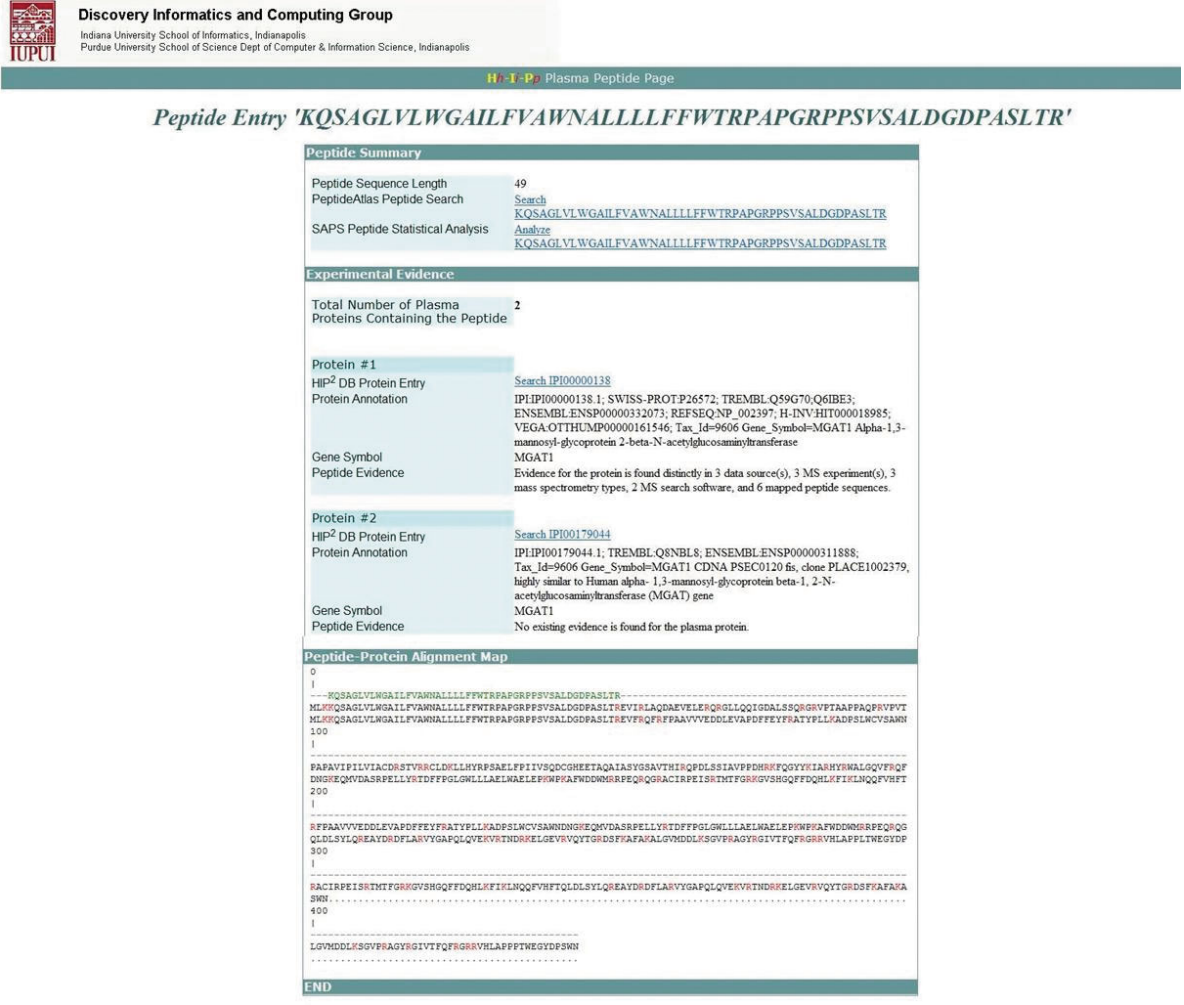
Snapshot of the peptide query search.

## Conclusion

The primary goal of the HIP^2 ^database is to support future clinical proteomics research, especially the discovery of biomarkers through plasma proteomics profiling. For biomedical researchers interested in MS-based plasma biomarker studies, HIP^2 ^can be the first database to search against a list of candidate proteins/genes or peptides before choices of prioritized biomarker candidates are made. As the database grows, additional annotation information of human plasma proteins such as relative abundance, normal range of variability, detectability, peptidomic patterns associative with cleavage, mutation and putative sites of glycosylation will be added. HIP^2 ^helps provide an integrated interface where database curators and data contributors can work together to collect ongoing published data from healthy human plasma proteomics experiments, foster community-based assessment of presence and absence of proteins in healthy human plasma, and provide a centralized data repository for subsequent bioinformatics analysis of the consistency and biases of each MS proteomics platform or search software. We expect this database to become an essential clinical proteomics resource, helping link together the community of biomedical researchers engaged in biomarker studies and the community of mass spectrometry researchers developing sensitive analytical solutions.

## Availability and requirements

The online content of HIP^2 ^is freely available to all WWW users. The database infrastructure and software tools used to develop the database are subject to the intellectual property protection terms of Indiana University.

Project name: Database for Healthy Human Individual's Integrated Plasma Proteome

Project home page: HIP^2 ^website [17]

Browser requirements: Modern browsers (e.g., Firefox or Microsoft Explorer) will function satisfactorily.

## Competing interests

The authors declare that they have no competing interests.

## Authors' contributions

JYC conceived the idea and constructed the general design of the database. SS collected data. SS and SHH processed data for insertion into an integrated database. SS and JYC built the web interface, and online documentation was written by RJA and JYC. SS, SHH and CS performed the statistical analyses and wrote the paper. RJA and XZ resolved issues with describing and categorizing MS platform technology. HT and PR helped characterize the analytical scope of peptide-based detection methodology. All authors participated with overall planning, editing and reviewing of the manuscript. All authors read and approved the final manuscript.

## Pre-publication history

The pre-publication history for this paper can be accessed here:


